# Strengthening Governance and Institutional Capacity at the Nutrition-Food Supply Policy Nexus: Comment on "What Opportunities Exist for Making the Food Supply Nutrition Friendly? A Policy Space Analysis in Mexico"

**DOI:** 10.34172/ijhpm.2022.7325

**Published:** 2022-08-01

**Authors:** Sirinya Phulkerd

**Affiliations:** Institute for Population and Social Research, Mahidol University, Nakhon Pathom, Thailand.

**Keywords:** Food System, Food Supply Chains, Nutrition, Governance, Mexico

## Abstract

Cervantes et al have provided an insightful addition to the policy literature by identifying the contextual, political, and policy factors that create constraints and opportunities for putting nutrition at the center of the food supply chain policy process. This commentary discusses important elements and features when aiming for reconciling nutrition goals and food supply policy, provides some examples of the salience of nutrition of non-health policies in countries with different income levels, and argues for improving governance for better nutrition outcomes and inspiring institutional interest and idea of food supply policy actors around population nutrition. Cervantes et al highlight the political context that favors nutrition outcomes, nutrition advocacy in the public agenda, and multisectoral mechanisms which can keep the nutrition objective moving forward in the food supply sector. However, a wider view on governance and institutional capacity is needed, recognizing government action by multiple sectors, with diverse sets of actors. The expanded understanding of nutrition, which includes considering nutrition as an emerging facet of food systems, by policy actors is needed. Enhancing discourse involving nutrition and food supply actors is important in order to appeal to the wider public and opinion leaders across the political spectrum. Accomplishing this also requires political will and an advocacy movement, especially by civil society and grassroots movements.

 Congratulations to Cervantes et al^1^ for their significant contribution to the food policy and systems literature in identifying opportunities and constraints to integrate nutrition goals into food supply policy in low- and middle-income countries, using a case study of Mexico. This study drew upon existing challenges in multisectorally addressing malnutrition, which have been globally recognized. The study combined political science concepts of policy space analysis an Advocacy Coalition Framework to shed light on the challenges and the opportunities faced by the government in integrating nutrition objectives into food supply policy. The policy space analysis was used to analyse the interrelation between context, agenda setting circumstances, and policy characteristics for opportunity for policy changes. The Advocacy Coalition Framework focused on actor dynamics in utilising strategies and resources to create policy changes. These theories and findings from the case study can be applied elsewhere to assess their relevance in other contexts. The study found that the economic issue is driven particularly by increased productivity and exportation as the major concerns in the food supply. Although nutrition was acknowledged (through food security) as important in the food supply chain, the focus was on food quantity, rather than dietary quality.

## Institutional Interests in Nutrition Issues

 Cervantes et al^1^ found that national advocacy coalitions in food supply play a prominent role within the nutrition and health landscape. Nutrition sits at the intersection of the food supply coalition (agriculture and economic sectors) and nutrition coalition (health sector). Promoting nutrition through food security was acknowledged to be an integral part of both of these food supply sectors. However, nutritional quality was not yet explicitly addressed. Priority in these sectors was given to sufficient quantity over sufficient quality. This is similar to the case of food supply policy in South Africa. The government has an implicit focus in food security related policies on food quantity, rather than nutritional quality. Nutrition and food security objectives were not supported by food supply policy actors.^2^

 The actors in the food supply chain are overlooking the true concept of food security, as defined by the World Food Summit (1996)^3^ as follows: “*Food security exists when all people, at all times, have physical and economic access to sufficient, safe, and nutritious food that meets their dietary needs and food preferences for an active and healthy life.”* If food security is not addressed explicitly, nutritional quality often gets lost between the food supply and nutrition policy sectors. This shortcoming speaks to the importance of clear, expanded understanding of nutrition, which includes a consideration of nutrition and health as an emerging facet of many societal systems, especially food systems among food supply actors. The lack of this understanding can undermine the translation of policy into more substantive political commitments in the food supply sector and, thus, limits the potential for achieving nutrition goals.

## Silos in Government

 Building greater integration of nutrition on the multisectoral policy space is important for pursuing a food system transformation to deliver sustainable healthy diets. This aspect is highlighted by Cervantes et al^1^ in that policies and initiatives, especially in the health and non-health sectors in low- and middle-income countries, suffer from having been primarily developed within “*practice silos,”* ie, through a piecemeal approach. Thus, those policies do not always consider the big picture, and their potential to interact (or have unintended consequences) has been largely ignored. Some examples of the “*practice silos”* are non-communicable disease (NCD) prevention efforts which were typically developed independently from healthy diet or food safety and preservation activities,^4^ as well as economic development efforts which mainly focused on increased productivity through modern food production and processing.^5^ As is the case in any system, solutions to one issue may create new problems for another. With respect to food and health, for example, food safety measures to limit microbial growth, such as the introduction of salt or food additives, can pose NCD risks.^6^ Another example is promotion of technological innovation in food production and processing, and foreign direct food investment to accelerate national economic growth. That strategy can lead to replacement of traditional agriculture and healthy diets by high-energy-dense and nutrient-poor foods such as ultra-processed foods which are associated with increased risk of NCD.^7,8^ This action can also lead to a reduced number of farming communities, rural-to-urban migration, low-wage employment, and greater economic inequity.^9^

 The complexity of food supply chains, nutrition, and health issues, also acknowledged by Cervantes et al,^1^ often adds to the challenge in dismantling the practice silos, promoting more collaboration, and creating synergies within the nutrition and food systems. A lack of recognition of nutrition and its importance in effective food supply chains can impede nutritional goals in other sectors.^10,11^ The need is for developing coordinative and communicative discourse on the issue of nutrition with policy actors in different contexts to more effectively address nutrition as a policy and development issue. The way these interactive processes are managed can determine whether policy actors feel motivated or threatened by articulating the linkage between the food supply chain and nutrition to the wider public and political actors.

 This is the case in Australian trade policy where nutrition has low salience, partly because of the complexity of nutrition and its inter-connection with trade, and how that presents difficulties for developing a broader discourse for engaging the public and political leaders on the issue.^11^ The communicative discourse on implications of trade policy was obtained by a focus on simpler health issues such as pharmaceuticals, and the impact which consumers experienced directly. There is also the case of investment policy in Thailand. Although better nutrition and economic progress are part of national development goals, as reflected in the 20-year National Strategy (2018-2037),^12^ challenges remain in reconciling the economic investment objectives of the strategy with population nutrition objectives. Thai investment policy still emphasizes promotion of “modern food production” and “foreign direct food investment” to add significant value to Thai products and production processes and, thus, increase productivity and accelerate economic growth.^13^

 Another case is the trade and investment policy in South Africa. Previous research has indicated that food supply chain was subject to binding international trade and investment agreements which constrain government policy space for uptake of nutrition policies.^14^ There has been increased trade and investment by processed food manufacturers and modern food retailers^15,16^ that become increasingly dominant as production and distribution channels for ultra-processed foods and beverages, associated with increased risks of diet-related NCDs.^17^ This suggests that significant gaps remain, and that there is a need for “whole-of-government” and “whole-of-society” approaches to facilitate joint government action, improve coordination, achieve integration, and devolve responsibility for nutrition and health throughout the various levels of government and society.

## Engagement of a Diverse Range of Actors

 Cervantes et al^1^ identified the steps of the food supply chain (production, distribution, transformation, markets) based on the food systems framework, developed by the *High Level Panel of Experts on Food Security and Nutrition of the Committee on World Food Security* (HLPE).^18^ For the purpose of their research, they placed conditions on these steps as part of the comprehensive food supply chain, and limited the scope and definition of food supply policy and the selection of appropriate sectors. While the food supply chain is one of three core constituent elements of food systems (as identified in the HLPE), there are other equally important influences which drive the food supply chain, thus shaping population diets and impacting on ultimate nutritional and health outcomes. In particular, changes to the food supply chain are largely influenced by biophysical, environmental, innovation, technology, infrastructure, political, and economic drivers.^19^ Therefore, the food supply chain connects a more diverse set of actors - both public and private - and more action and policies need to be considered, especially in the spheres of natural resources, science, finance, foreign investment, trade, urban planning, and humanitarian sectors. Such a broad approach will provide greater insight for the present study if actors in these sectors are involved in the analysis.

## Power of Short Food Supply Chains

 Cervantes et al^1^ outline important strategies for aligning the food supply with desired nutritional outcomes in a country. *Shortening food supply chains* (SFSC) was recognized by many actors as an important strategy since it has high potential to reconnect farmers with consumers. This is especially important for smaller, lower-income farmers who have trouble equitably accessing global markets.^20,21^ Creating this linkage can contribute to improving food and nutritional security for consumers as well as farmers, their families, and host communities. The benefits of the SFSC are considerable, and range from economic to social, environmental, nutritional and local development aspects, for both producers and consumers.^20^ The reduction of distances allows the value of local production to remain within a small community, and not dispersed along extensive distribution channels that take food products far from the farm.^21^ Producers can communicate directly with consumers, and vice versa, easing the flow of information about product, process, and participants. This approach can strengthen social ties, build solidarity, and boost awareness, thus enhancing the local economy. Consumers are able to exercise greater control over what they purchase, eg, by buying farm-to-table products which are generally fresher and more diversified at lower prices, with the added benefit of positive impact on their health and, at the same time, contribute to strengthening local food networks.^22^ The SFSC strategy also reduces the environmental impact of agriculture such as greenhouse gas emissions from food transport.^23^ In response to the 2030 United Nations (UN) Agenda for Sustainable Development, SFSC can contribute to multiple Sustainable Development Goals (SDGs), eg, SDG # 1, 2, 5, 8, 9, 11, 12 and 13.^24^

## Political Will as a Sine Qua Non for Policy Change

 Drawing on the results, Cervantes et al^1^ point to a window of opportunity for nutrition integration that comes from the political context and public agenda which favor a food system transformation toward nutrition and health outcomes. There is widespread recognition of the importance of political will in charting a path toward better alignment of agriculture, economic, nutrition, and health goals. For example, the World Health Organization (WHO) High-Level Commission on NCDs has suggested that in order to move forward to implementation the most obstacle to overcome is political. There must be the political will to do so,^25^ and that a sufficient set of decision makers with a common understanding of a particular problem is needed. However, a crucial issue is how to enable key actors and politicians to embrace this mindset, especially by putting their rhetoric into action. The challenge is to balance nutrition/health and economic objectives in policymaking. Political will requires a shift toward norms which see food insecurity and poor nutrition as a result of “broken” food systems. At present, too many systems are exacerbating the double burden of under- and over-nutrition, leading to the degradation of ecosystems, and driving many farming families into poverty.^26^ Creating the political will to challenge unhealthy policies will require politically-led, institutional change to align investment in the food system with other social and health goals, that agricultural and industrial policies often ignore.^27^

## Incentive Measures for Private Sector

 Private sector actors play an important role, as they are key investors in the food system. Innovative investment incentives and mechanisms to support the adoption of healthy food supply practices and improve productivity, therefore, can provide an attractive basis to encourage private sector investment for sustainable and healthy food systems.^27^ Thus, it is critical for the government to find ways to incentivize the private sector to include improved nutrition among its goals. This also involves balancing nutritional needs with commercial interests, and managing conflicts of interest, and that will require participatory and transparent governance. This position is likely to have broad appeal if backed by strong political leadership and a popular advocacy movement, especially propelled by civil society and grassroots mobilization that can provide local help, and push government to change. At the very least, this advocacy movement should result in raising awareness of incompatible partnerships and undue corporate interference in the policy formulation process.

 When aiming for improving nutrition and health in food supply space, it requires good governance and institutional capacity. The governance and institution should recognize government action by different actor groups. It is important to expand understanding of nutrition, especially linking food supply with nutrition and health outcomes, by food supply policy actors. The governance necessitates communicative and coordinative discourses between nutrition actors and food supply actors to increase opportunities for reconciling nutrition goals in food supply policy. Accomplishing this also requires political will and advocacy movement as a sine qua non for making food supply nutrition friendly. The government should also create innovative incentives for the private sector to include improved nutrition among its goals. Learning from other countries’ experiences and international comparisons can be useful.

## Ethical issues

 Not applicable.

## Competing interests

 Author declares that she has no competing interests.

## Author’s contribution

 SP is the single author of the paper.
